# The Multifunctional Protein Kinase C-ε in Cancer Development and Progression

**DOI:** 10.3390/cancers6020860

**Published:** 2014-04-10

**Authors:** Kirti Jain, Alakananda Basu

**Affiliations:** Department of Molecular and Medical Genetics, University of North Texas Health Science Center, Institute for Cancer Research, and Focused on Resources for her Health Education and Research, Fort Worth, TX 76107, USA; E-Mail: kjain@live.unthsc.edu

**Keywords:** PKCɛ, survival, EMT, metastasis, cancer stem cells, microRNA

## Abstract

The protein kinase C (PKC) family proteins are important signal transducers and have long been the focus of cancer research. PKCɛ, a member of this family, is overexpressed in most solid tumors and plays critical roles in different processes that lead to cancer development. Studies using cell lines and animal models demonstrated the transforming potential of PKCɛ. While earlier research established the survival functions of PKCɛ, recent studies revealed its role in cell migration, invasion and cancer metastasis. PKCɛ has also been implicated in epithelial to mesenchymal transition (EMT), which may be the underlying mechanism by which it contributes to cell motility. In addition, PKCɛ affects cell-extracellular matrix (ECM) interactions by direct regulation of the cytoskeletal elements. Recent studies have also linked PKCɛ signaling to cancer stem cell functioning. This review focuses on the role of PKCɛ in different processes that lead to cancer development and progression. We also discussed current literatures on the pursuit of PKCɛ as a target for cancer therapy.

## 1. Introduction

Kinases are central mediators of signal transduction and have become attractive targets for drug development [1]. An important class among them is protein kinase C (PKC) which constitutes 2% of the human kinome [2]. PKC is a family of structurally related serine/threonine kinases that are classified as conventional, novel and atypical based on their structural properties and responsiveness to second messengers [3,4,5,6,7]. Conventional PKCs (PKCα, -βI, -βII and -γ) contain two diacylglycerol (DAG)-binding domains (C1A and C1B) and a calcium-binding domain (C2) and therefore are responsive to both DAG and calcium [5,8,9]. Novel PKCs (PKCδ, -ɛ, -η and -θ) contain a variant of C2 domain (C2-like domain) and do not require calcium for activation [9,10]. Atypical PKCs (PKCζ and –I/ι) lack functional binding sites for DAG as well as calcium and are therefore independent of both for activation [9,11,12,13].

Since their discovery as receptors for tumor-promoting phorbol esters, PKCs have been intensively studied for their contribution to cancer [14]. Common processes regulated by PKCs include cell survival, proliferation, apoptosis, migration and invasion [9]. PKCs can have similar, overlapping and sometime opposing roles in cellular processes [15]. Moreover, the function of most PKCs in cancer has been found to be dependent on the cellular context. PKCɛ is the first isozyme that was shown to possess oncogenic functions and is emerging as an undisputed tumor promoter [16].

PKCɛ was identified as a novel PKC isotype by cDNA cloning from rabbit and rat brain [17,18]. More than two decades of research on PKCɛ has shown it to be a dynamic player in diverse cellular processes. At the systemic level, PKCɛ activation has protective roles in cardiac and brain ischemia, nociception and heat shock response while uncontrolled PKCɛ activation is associated with cancer development [19,20]. PKCɛ is overexpressed in various tumor types [16] and is associated with different processes related to cancer development namely, cell transformation, cell survival, cell proliferation, EMT, cytoskeletal reorganization, extracellular matrix (ECM) rearrangement, disruption of cell-cell contacts, cell motility, stem cell properties and therapy resistance [16,19,21,22]. In this review article, we primarily focused on the salient functions of PKCɛ in cancer development and progression, and its potential as a target for cancer therapy with an emphasis on the recent contributions.

## 2. PKCɛ in Oncogenic Transformation

One of the first evidence demonstrating the oncogenic potential of PKCɛ came from studies of Mischak *et al.* [23]. The authors demonstrated that overexpression of PKCɛ in NIH 3T3 murine fibroblasts showed transformed phenotype as evident from increased growth rates in cell culture and in soft agar, as well as increased tumor incidences in xenograft models. Similarly, PKCɛ was found to be oncogenic in rat 6 fibroblasts [24] and rat colonic epithelial cells *in vitro* and *in vivo* [25]. PKCɛ-overexpressing rat colonic epithelial cells showed Raf-1/mitogen activated protein kinase (MAPK) to be responsible for the PKCɛ-induced transformation [26].

Generation of transgenic mice models with tissue-specific overexpression of PKCɛ has been achieved for skin and prostate tissues [27,28]. Although there was no noticeable difference between parental and transgenic mice overexpressing PKCɛ in skin epidermis, exposure to ultraviolet radiation (UVR) resulted in increased incidences of squamous cell carcinoma in PKCɛ mice signifying a role for PKCɛ in skin cancer development [28]. Recently, Kazanietz and co-workers generated transgenic mice models for prostate-specific expression of PKCɛ, -α and -δ [29]. In this study, PKCɛ mice developed hyperplasia and prostate intraepithelial neoplasia (PIN) which were not observed in the wild-type control, PKCα or PKCδ mice [29,30]. These studies suggest a causal role for PKCɛ in tumor initiation. In the prostate specific transgenic mouse model, phosphorylation of the serine/threonine kinase Akt and signal transducer and activator of transcription 3 (Stat3) was found to be increased [29]. Similarly, PKCɛ increased UVR-induced phosphorylation of phosphoinositide 3-kinase (PI3K), Stat3 and extracellular signal-regulated kinase (ERK) in mouse model of skin cancer [28]. Thus, PI3K/Akt, Stat3 and MAPK/ERK pathways are the likely mediators of PKCɛ-induced transformation.

## 3. PKCɛ in Cell Survival

It is well-established that PKCɛ promotes cell growth and functions as an anti-apoptotic protein. It inhibits both pathways of apoptosis—the mitochondrial or intrinsic pathway and the receptor-mediated or extrinsic pathway [21]. It cooperates with different signaling pathways to promote cell survival [21]. The prominent survival pathway activated by PKCɛ is Akt. PKCɛ can phosphorylate Akt directly [31] or indirectly via other kinases [21,32,33]. PKCɛ-mediated Akt phosphorylation/activation positively regulated cell survival in different cellular contexts as reviewed earlier [21].

Nuclear factor кB (NF-кB) is another important oncogenic pathway that is activated downstream of PKCɛ [34,35]. PKCɛ mediated activation of NF-кB promoter in rat fibroblasts [35]. Transgenic mice with prostate-specific overexpression of PKCɛ developed preneoplastic lesions that displayed hyperactivation of NF-кB [34]. PKCɛ also mediated tumor necrosis factor α (TNFα)-induced NF-кB activation by facilitating the assembly of TNF receptor-1 signaling complex in prostate cancer cells [34]. The study by Yang *et al*. provided mechanistic insights into the PKCɛ-mediated activation of NF-кB downstream of epidermal growth factor receptor (EGFR) in glioblastoma cells [36]. Phospholipase C γ-1 activation, in response to EGF, resulted in monoubiquitylation of PKCɛ. Docking of NF-κB essential modulator (NEMO) on to the monoubiquitinated PKCɛ led to the recruitment of inhibitor of кB kinase (IKK) complex to the membrane and subsequent phosphorylation of IKKβ by PKCɛ [36]. The resulting NF-кB activation caused transcriptional induction of pyruvate kinase M 2 (PKM2) which facilitated glycolysis and mediated development of glioblastoma multiforme [36].

PKCɛ promotes cell survival not only by activating survival pathways but also by inhibiting pro-apoptotic signaling. Important in this regard is the regulation of Bcl-2 family members by PKCɛ. We and others have previously shown that PKCɛ increased the levels of anti-apoptotic Bcl-2 protein in different cell types [37,38]. In addition, PKCɛ was shown to increase anti-apoptotic Bcl-X_L_ and X-linked inhibitor of apoptosis protein (XIAP) and to decrease proapoptotic BH3 interacting-domain death agonist (BID) [37,39,40,41]. PKCɛ inhibited Bax activation by blocking its translocation to mitochondria [42] while it inhibited Bad by increasing its phosphorylation at Ser112 [43,44]. Thus, PKCɛ inhibits apoptosis by regulating the levels, phosphorylation status or localization of Bcl-2 family proteins.

New PKCɛ targets, which have important roles in mitochondria, have been discovered. PKCɛ-mediated Stat3 activation increased mRNA and protein levels of translocator protein TSPO [45], a cholesterol- and drug-binding protein that is primarily located at the outer mitochondrial membrane [46]. Modulation of TSPO levels may have important implications in the regulation of mitochondrial apoptosis by PKCɛ. Another important target of PKCɛ is the dual function protein ATF2 (activating transcription factor 2) [47]. ATF2 functions as an oncogene in melanoma but as a tumor suppressor in non-malignant skin cancer [47]. Lau *et al*. showed PKCɛ to be a decisive factor in ATF2 functioning [47]. ATF2 is a stress-induced protein which, in response to genotoxic agents, can translocate to mitochondria and mediate membrane permeabilization by direct interaction with hexokinase-1 and voltage-dependent anion channel 1 (VDAC1) [47]. However, ATF2 phosphorylation at Thr52 by PKCɛ prevents its mitochondrial translocation and directs it to the nucleus, thereby attenuating apoptosis in response to genotoxic drugs [47]. Moreover, inhibition of PKCɛ-mediated ATF2 phosphorylation resulted in cytoplasmic targeting of ATF2 and reduced oncogenic properties of melanoma cells [49]. Thus, high levels of PKCɛ in melanoma are responsible for promoting chemoresistance and tumorigenesis by nuclear targeting of ATF2 [48,49].

## 4. PKCɛ in Metastasis

The primary cause of morbidity among cancer patients is metastasis [50]. PKCɛ is associated with metastasis and aggressive phenotype in most cancers [16]. In breast tumor samples, PKCɛ overexpression was associated with high histologic grade, positive Her2 status and negative estrogen and progesterone receptor status [51]. Its expression was found to be a predictor of poorer overall and disease-free survival in breast cancer patients [51]. Conversely, its depletion led to a less aggressive phenotype *in vitro* as well as in xenograft models [51,52]. PKCɛ expression also correlated with tumor grade in prostate tumor samples [53]. Overexpression of PKCɛ transformed androgen-dependent prostate cancer cells into androgen-independent type and led to the formation of aggressive tumors when transplanted into nude or castrated mice [54]. PKCɛ was also shown to be overexpressed in early prostate adenocarcinomas [55]. Additionally, PKCɛ overexpression was associated with poor prognosis in head and neck squamous cell carcinoma patients [56,57] and its depletion resulted in less motile phenotype *in vitro* [57].

Among the cancers of urogenital tract, PKCɛ expression correlated with tumor grade and stage in clear cell renal cell carcinoma (RCC) and its depletion resulted in decreased cell growth and migration in RCC cells [58]. In cancers of the nervous system, PKCɛ showed elevated expression in astrocytoma, glioblastoma multiforme and gliosarcoma tumor samples [59] as well as in glioblastoma cell lines [60]. PKCɛ is also overexpressed in non-small cell lung carcinomas (NSCLC) [61]. Inhibition of PKCɛ using dominant-negative mutant resulted in reduced aggressiveness of the NSCLC cells as measured by the decrease in proliferation and anchorage-independent growth [61]. Thus, PKCɛ is associated with aggressive phenotype in most solid tumors and is considered to be a biomarker for metastatic cancers [16].

There is limited information on the mechanisms responsible for observed overexpression of PKCɛ in cancers. A number of tumor samples containing amplifying somatic mutations in PRKCE gene are listed in the COSMIC database [62]. In addition, recent studies have shown PKCɛ to be a target for a number of tumor-suppressor microRNAs (miRNA). PKCɛ is targeted by miR-205 in prostate cancer [63], miR-107 in head and neck squamous cell carcinoma [64], miR-31 in breast cancer [65], miR-143 in lung cancer [66] and miR-146a in papillary thyroid cancers [67]. Moreover, PKCɛ was functionally validated to be a downstream target of these miRNAs in respective tissues/cells [63,64,65,66,67]. PKCɛ is also a target of miR-129 in lung epithelial cells [68] although the functional significance of this regulation is not known. Thus, microRNAs may be an important means of regulating PKCɛ expression and their downregulation may explain, in part, the increased expression of PKCɛ in cancers.

## 5. Regulation of EMT by PKCɛ

Cancer metastasis involves a series of steps starting with dissemination of cells from the primary tumor, migration and invasion through the stroma, intravasation into the blood vessels, anoikis resistance and circulation through the blood stream, extravasation from the blood vessels and finally formation of tumor at the secondary site [69]. Most solid tumors arise from epithelial cells which are characterized by cuboidal shape, cell-cell and cell-matrix adhesion and apico-basal polarity [70]. In order to migrate to a distant site, the epithelial cells lose their epithelial characteristics and gain mesenchymal features like spindle shape and increased migratory and invasive potential [71]. This metamorphosis of epithelial cells to mesenchymal phenotype is called epithelial to mesenchymal transition or EMT [72]. EMT was first studied as a phenomenon essential during embryonic development, by which the epithelial cells from the primary tissue migrate to a different site [73]. Cancer cells, however, hijack this process to migrate to distant sites during metastasis [74].

The phenotypical changes in EMT are associated with a corresponding change in molecular markers such as the loss of adhesion proteins (e.g., E-cadherin, Zonula Occludens-1 or ZO-1 and claudins) and gain in proteins abundant in mesenchymal cells (e.g., vimentin, N-cadherin and fibronectin) [72]. These drastic changes in cell’s cytoskeleton require a major transcriptional reprogramming. Prominent inducers of EMT *in vitro* and *in vivo* are transforming growth factor (TGF)-β and bone morphogenic protein (BMP) [75]. In addition, other growth factors like epidermal growth factor (EGF), platelet derived growth factor (PDGF) and cytokines can promote EMT in a context-dependent manner [76,77,78,79]. Recent studies have shown a crucial role for PKCɛ in EMT and cell migration [51,63,80,81].

The change of cell morphology from cuboidal to spindle type is the most striking feature of EMT. The propensity of PKCɛ to change cell morphology was originally identified by Parletti *et al.* [25,82]. Subsequently, several reports demonstrated the role of PKCɛ in the regulation of cytoskeleton and cell migration [16,83,84,85,86,87]. Gandellini *et al*. showed that PKCɛ is an important target of miR-205, and depletion of PKCɛ was associated with reversion of the aggressive prostate cancer cells to epithelial type as determined by increased E-cadherin expression and decreased cell motility [63]. We recently showed that overexpression of PKCɛ was sufficient to induce EMT in non-malignant breast epithelial MCF-10A cell line [80]. In addition, PKCɛ promoted anoikis resistance and cell migration that are important characteristics of EMT [80]. Moreover, depletion of PKCɛ led to partial reversion of TGFβ-induced mesenchymal phenotype [80]. These studies demonstrate an important role for PKCɛ in EMT.

While the precise mechanism by which PKCɛ promotes EMT has not been delineated, there are a number of interesting possibilities. Firstly, PKCɛ was shown to mediate phosphorylation of vimentin, an intermediate filaments that is increased in mesenchymal cells [85]. Vimentin phosphorylation was required for vesicular trafficking and directional cell motility towards the matrix [85,88]. A recent report showed the involvement of PKCɛ in mediating phosphorylation of ZO-1 at Thr770/Thr772 residues [89]. Thr770/Thr772 phosphorylation of ZO-1 was associated with disruption of tight junctions in endothelial cells [89]. PKCɛ is also known to phosphorylate and thereby cause nuclear export of ZO-2 [90] although its functional significance is not known. Whether regulation of vimentin and/or ZO-1/2 by PKCɛ is sufficient to cause EMT needs to be explored. An earlier report showed that overexpression of PKCɛ in rat fibroblasts increased the production of active TGFβ [91], a leading inducer of EMT *in vitro* and *in vivo* [75]. Thus, PKCɛ may induce EMT via TGFβ in an autocrine manner. TGFβ signals through Smad receptors and leads to the upregulation of EMT-inducing transcription factors (e.g., Snail, Slug and Twist), prominent among them being Snail [75,92,93]. We showed that the reversal of TGFβ-induced mesenchymal phenotype in PKCɛ-depleted cells was associated with a concomitant decrease in Snail levels [80]. Thus, PKCɛ and TGFβ may operate in a positive feedback loop to bring about the mesenchymal phenotype.

PKCɛ also increased the production of some cytokines and growth factors via TNFα-converting enzyme (TACE) [94,95]. TACE is a member of the matrix metalloprotease (MMP) family that mediates the ectodomain shedding of many secreted proteins [53]. PKCɛ caused phosphorylation and activation of TACE and thereby increased the shedding of TNFα and amphiregulin (a member of the EGF family of ligands) [94,95]. Signaling downstream of TNFα as well as EGF receptor pathway are known to cause EMT in specific contexts [76,78]. IL-6 is another cytokine that is increased by PKCɛ [63,96] and can induce EMT [79,97]. In addition, PKCɛ can promote autocrine signaling of fibroblast growth factor (FGF) [98], another well-known EMT mediator [72,99]. PKCɛ can also phosphorylate FGF receptor (FGFR) at Ser779 and thereby promote its downstream signaling [100]. Thus PKCɛ-mediated cytokine and growth factor signaling may, in part, be responsible for EMT induction.

The signals emanating at the cell surface, in response to growth factors and cytokines, are usually transduced via activation of different intracellular kinases and transcription factors. The most important serine/threonine kinase that is linked to PKCɛ signaling is Akt [21]. There are three isoforms of Akt namely Akt1, Akt2 and Akt3 [101]. We and others have previously shown that PKCɛ promotes apoptosis-resistance via activation of Akt1 [21,32,102,103]. While Akt1 is mostly involved in cell survival and proliferation, Akt2 plays a role in EMT induction and cell migration [104]. It is possible that PKCɛ recruits Akt2 to promote EMT.

Among the transcription factors, Stat3, a substrate of PKCɛ, also has a role in EMT induction [81,96,105]. PKCɛ activates Stat3 by direct phosphorylation at Ser727 residue and this regulation is important for cell invasion and motility [53,81]. Moreover, Stat3 mediates TGFβ-induced EMT by transcriptional upregulation of Twist [105,106]. LIV1 is another Stat3 target, which is required for the nuclear translocation of Snail [107]. Thus, it is possible that TGFβ, PKCɛ and Stat3 collaborate to induce EMT.

One important class of proteins connecting various signaling hubs inside the cell is Rho family of GTPases [108]. Rho A and Rho C have been implicated in regulating cell motility downstream of PKCɛ [51]. Particularly, PKCɛ was shown to phosphorylate Rho A at Thr127 and Ser188 sites [109]. In addition, Rho A and Rho C are known to promote EMT [109,110,111]. Thus Rho GTPases may facilitate signal transduction through PKCɛ to bring about EMT.

In summary, there are a number of PKCɛ targets that participate in EMT. These different EMT-mediators may form one linear axis or work through divergent pathways in different cellular contexts.

## 6. PKCɛ in the Regulation of Cytoskeleton

In addition to the regulation of EMT, PKCɛ can directly interact with and regulate cytoskeletal elements and thereby participate in cell-ECM interactions. An earlier study showed that PKCɛ is the only PKC isozyme that translocates from the cytosol to the plasma membrane during cell adhesion and spreading to a gelatin matrix [112]. An important interacting partner of PKCɛ during cell adhesion is the transmembrane adhesion molecule β1 integrin [113]. Adhesion and migration of cardiac fibroblasts required PKCɛ-mediated phosphorylation of Thr788/Thr789 residues in the cytoplasmic tail of β1 integrin [113]. Likewise, PKCɛ was required for the lamella formation during migration of lung cancer cells [87]. PKCɛ localized to the leading edge of the migrating cell and controlled the lamella formation by promoting a complex formation between tight junction protein ZO-1 and α5β1 integrin [87]. In addition, PKCɛ mediated vesicular trafficking of β1 integrin which was necessary for directional cell migration [88]. Thus, PKCɛ is intimately connected to integrin signaling and this cross-talk forms critical component of the cell migration machinery.

A unique feature of the PKCɛ structure is its ability to bind actin [19]. The 223–228 hexapeptide (LKKQET) in the conserved C1 domain of PKCɛ constitutes the actin binding motif and remains unexposed in the inactive conformation of the protein [114]. Active PKCɛ binds to filamentous actin and this interaction stabilizes the active conformation of PKCɛ [114]. Since actin cytoskeleton remodeling and dynamic formation of focal adhesions is crucial for cell motility, it is hypothesized that PKCɛ participates in cell migration by promoting F-actin assembly [59]. Likewise, PKCɛ translocation to focal adhesions was required for phorbol myristate acetate (PMA)-induced migration in glioma cells [115]. In neuronal cells, PKCɛ-mediated actin polymerization was involved in neurite outgrowth and glutamate exocytosis [84,116]. It is conceivable that PKCɛ is involved in the formation of long protrusive processes in different cellular contexts be it neurite outgrowth or cancer cell migration.

An indirect but a critical regulation of the cytoskeleton by PKCɛ is demonstrated during cytokinesis [117]. Phosphorylation of PKCɛ at three sites (Ser350, Ser346 and Ser368) leads to its binding with 14-3-3 [117,118]. The PKCɛ-14-3-3 complex is required for the final abscission step in cytokinesis [119]. Rho A GTPase helps with the contraction of actomyosin ring that creates the furrow between the two poles of a dividing cell [119]. However, inhibition of Rho A activity is implicated in the final “pinching off” of two daughter cells. It is suggested that PKCɛ-14-3-3 complex inhibits Rho A and thereby brings about the abscission step of cytokinesis [117,119]. Another study showed that PKCɛ-dependent phosphorylation of ZO-1 at Ser168 is required for the completion of cytokinesis [120].

An important downstream target of PKCɛ is caveolin [121]. Caveolins are transmembrane adaptor proteins that participate in receptor-independent endocytosis [122,123]. Although traditionally known to be a tumor suppressor, caveolin-1 has recently been implicated in cancer development [124]. Caveolin-1 is overexpressed, and is associated with disease aggressiveness in prostate cancer [125]. PKCɛ was shown to increase the expression and secretion of active caveolin-1 in recurrent prostate cancer cells [121]. Further research is needed to determine if PKCɛ regulates cell-matrix interactions via caveolin-1.

## 7. PKCɛ and Cancer Stem Cells

Development of different functional assays like clonogenic assay, transplantation and lineage tracing and state-of-the-art cell isolation methods have helped ascertain the existence of stem cells and reveal the hierarchical nature of cells in tissues [126,127]. Similar heterogeneity of cells in tumor, and multistep model of oncogenesis, formed the basis for cancer stem cell (CSC) model [126]. Based on the CSC model, the tumors are initiated and prorogated through a malignant population of cells that share with normal stem cells the ability to renew infinitely and to differentiate into other cell types [126]. As such, CSCs are supposed to be the key cause of therapy resistance and tumor recurrence. Complete eradication of the cancer stem cells would therefore be required for effective treatment of cancers [127]. Consequently, there is a mounting interest in understanding the signaling networks that sustain CSCs.

Several studies have linked PKCɛ signaling to cancer stem cells. One of the organs best studied for tissue hierarchy and stem cell lineage is colon. A colon crypt usually contains a small population of stem cells at the bottom while the sequentially more differentiated cells are located towards the top of the crypt [128]. Gobbi *et al*. showed that PKCɛ expression is highest at the bottom of the colon crypt and decreases gradually away from the crypt [129]. PKCɛ protein expression also varied inversely with TRAIL (TNF-related apoptosis-inducing ligand) which promoted cell differentiation in the colon [129]. Moreover, downregulation of PKCɛ promoted differentiation of stem cells in culture [129]. In addition, PKCɛ inhibited the differentiation of neural stem cells [130] while promoted the survival of glioma stem cells [40]. These studies implied a role for PKCɛ in maintaining a pool of undifferentiated stem cells.

Singh *et al.* directly addressed the effect of PKCɛ on stem cells population in hair follicles [131]. They showed that PKCɛ overexpression in mice increased the number of double positive (CD34+/α6-integrin+) hair follicle stem cells (HSCs) in response to UV radiation (UVR) [131]. The HSCs in transgenic mice also cycled at a faster rate and displayed increased expression of genes that are involved in transformation, invasion and metastasis [131]. It is known that PKCɛ mice develop metastatic skin cancer in response to UVR [28]. Thus, it is conceivable that PKCɛ promotes tumor malignancy by sustaining CSCs in UVR-induced skin cancer models.

A recent study identified PKCɛ as a downstream effector of hypoxia and stem cell factor (HASF), which is a stem cell paracrine factor [132]. On the other hand, PKCɛ directly regulated the embryonic stem cell marker Nanog. Bourguignon *et al*. showed that PKCɛ increased the phosphorylation of Nanog in MCF-7 breast cancer cells [22]. Nanog phosphorylation triggered its nuclear translocation where it regulated the processing of microRNAs [22]. In a later study, Piao *et al.* showed that Nanog is directly phosphorylated by PKCɛ at Thr200 and Thr280 residues [133]. Moreover, PKCɛ-mediated phosphorylation enhanced protein stability of Nanog and was required for its transcriptional activity [133]. Moreover, Nanog phosphorylation was required for supporting a population of cancer initiating cells (CICs) as overexpression of non-phosphorylatable mutant decreased CICs [133]. In addition, treatment of cancer cells with Pre-miR-107 containing nanoparticles resulted in decreased expression of PKCɛ as well as that of stem cell markers Nanog, Sox2 and Oct3/4 [134]. Furthermore, miR-107 treatment decreased CICs [134]. These studies suggest a role for PKCɛ in promoting the growth of CICs via activation of stem cell marker Nanog.

## 8. Therapeutic Targeting of PKCɛ

As PKCɛ is involved in various pathologies, its activators and inhibitors have been long sought after. High homology between different PKC isozymes prohibited the design of specific chemical inhibitors against PKCs. Modest selectivity has been achieved using short peptide inhibitors [135,136,137]. These peptides were designed to inhibit the interaction of PKCɛ with its adaptor proteins or to prevent its translocation to the membrane [135,136,137]. Bao *et al*. reported the design and the use of a novel bifunctional peptide HN1-PKCɛ in HNSCC [138]. HN1-PKCɛ was designed by linking the cancer cell homing (HN1) module with PKCɛ translocation inhibitory module (PKCɛ), so as to achieve inhibition of PKCɛ specifically in cancer cells [138]. This peptide preferentially penetrated HNSCC cells *in vitro* and *in vivo* and significantly retarded the growth of tumor xenografts in mice [138].

Another novel approach in PKCɛ activity modulation is the development of Llama single chain antibodies (denoted by VHH) [139]. VHH antibodies are much smaller and more stable than conventional antibodies [139]. In addition, they can recognize relatively eclipsed part of the enzyme [139]. Activating as well as inhibitory VHH antibodies have been made against PKCɛ and have been functionally validated in HeLa cells [139,140]. We expect that this antibody approach will be extended to different model systems and further modification of these antibodies will allow their use in the clinic.

## 9. Conclusions

**Figure 1:**
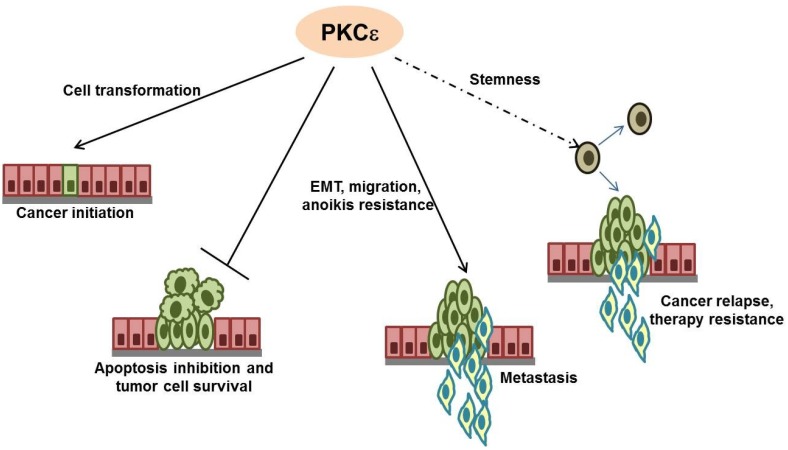
The involvement of PKCɛ in various steps of cancer development and progression.

More than two decades of research on PKCɛ has established this kinase to be a key player in different cancers. It is overexpressed in most solid tumors and is being increasingly shown to be a target of tumor suppressor microRNAs. Functionally, it plays crucial roles in almost all aspects of tumor development, namely cell transformation, proliferation, cancer cell survival, EMT, migration and invasion (Figure 1). 

In addition, it is recently implicated in cancer cell stemness. Thus, PKCɛ has emerged as an important candidate for cancer therapy. However, before PKCɛ can be targeted in the clinic, a clear understanding of its regulatory network is required. Moreover, the particular subsets of tumors, in which PKCɛ inhibitors may be used for mainstream or adjuvant therapy, need to be identified. Parallel efforts are also required to develop more specific pharmacological inhibitors of PKCɛ. Clinical use of PKCɛ inhibitors will also demand highly specific delivery to the tumor to avoid damage to other vital organs like brain and heart, wherein PKCɛ plays a protective role. Thus, with a collaborative multidisciplinary effort, clinical targeting of PKCɛ may ultimately be possible for the treatment of cancer.
